# Hyperspectral Image Classification Based on Improved Rotation Forest Algorithm

**DOI:** 10.3390/s18113601

**Published:** 2018-10-23

**Authors:** Fei Lv, Min Han

**Affiliations:** Faculty of Electronic Information and Electrical Engineering, Dalian University of Technology, Dalian 116085, China; lvfei@mail.dlut.edu.cn

**Keywords:** hyperspectral image classification, rotation forest, extreme learning machine, Q-statistic

## Abstract

Hyperspectral image classification is a hot issue in the field of remote sensing. It is possible to achieve high accuracy and strong generalization through a good classification method that is used to process image data. In this paper, an efficient hyperspectral image classification method based on improved Rotation Forest (ROF) is proposed. It is named ROF-KELM. Firstly, Non-negative matrix factorization( NMF) is used to do feature segmentation in order to get more effective data. Secondly, kernel extreme learning machine (KELM) is chosen as base classifier to improve the classification efficiency. The proposed method inherits the advantages of KELM and has an analytic solution to directly implement the multiclass classification. Then, Q-statistic is used to select base classifiers. Finally, the results are obtained by using the voting method. Three simulation examples, classification of AVIRIS image, ROSIS image and the UCI public data sets respectively, are conducted to demonstrate the effectiveness of the proposed method.

## 1. Introduction

Remote sensing technology is a non-contact and long distance detection technology. With the development of Internet of Things (IoT) technology [1,2], the field of remote sensing also shows new vitality, more and more remote sensing information can be obtained, such as low-resolution remote sensing images, hyperspectral remote sensing images and so on. IoT technology plays an important role in the process of remote sensing data acquisition. Abundant remote sensing information can also greatly improve the accuracy of remote sensing image classification, as well as people’s in-depth study of remote sensing images. The acquisition of ground image information by remote sensing technology is becoming more and more fine. Hyperspectral remote sensing images have been obtained by the way of the airborne instrument on the IoT [3,4,5]. The classification of hyperspectral remote sensing images has also become a hot topic for many scholars. The classification is a method to distinguish the property and distribution of ground objects according to the information characteristics of remote sensing image. It is an area worth exploring.In the field of remote sensing, the emergence of hyperspectral remote sensing image data classification technology is a revolution [6]. Generally, an algorithm is used for the classification of remote sensing image, such as decision tree, using the data of dimensionality reduction as input signals. These algorithms have proven their advantages in a lot of experiments, but it still exists some shortages. Firstly, single classifier has its limitations, and it cannot often get better classification accuracy for a single classifier. Secondly, hyperspectral remote sensing data has a great connection with adjacent bands, so all bands are not guaranteed high accuracy at the same time [7,8]. According to these limitations, some new methods are needed to improve the algorithm performance. On the basis of summarizing hyperspectral remote sensing classification technology and ensemble algorithm, this paper discusses the classification problem of hyperspectral image data based on ensemble method. Some researchers have proposed that an ensemble algorithm can deal with this issue. Chi et al. [9] proposed that an ensemble algorithm is used to deal with remote sensing image classification, and it has stability.

With the continuous development of IoT [10], remote sensing technology is constantly updated, and remote sensing image classification methods are also improving. The method of improving the classification effect of the integrated classifier is basically carried out in two aspects: the precision of the base classifier and the diversity between the base classifiers. So a key point needs to be solved, which is how to improve the diversity. To this point, Garcia-Pedrajas [11] set each base classifier the weights of each training phase. This method is affected by false index data and can lead to overfit. Rodriguez et al. [12] proposed an ensemble algorithm called Rotation Forest based on Random Forest. It is to improve the diversity of members and the precision of base classifier. Rotation Forest uses the decision tree method as each independent structure classifier, and the rotation of the principal component analysis (PCA) transform in the feature space for the training of the training sample. The most important point of the collection method is the selection of base classifier. The decision tree has been used to rotate tasks because of its sensitivity to the rotation of the characteristic axis. Lee et al. [13] demonstrated an algorithm for non-negative matrix factorization (NMF). For non-negative data, NMF achieves better results than PCA algorithm.

To deal with the issue of classification efficiency and accuracy, Huang et al. [14] proposed extreme learning machine(ELM) neural network. ELM is a new neural network training paradigm, where a non-iteration learning method is performed. ELM randomly generates the hidden layer parameters, and are independent of training error and output power. It has better generalization performance, and has a unified analytical solution for binary, multi-class and regression problems. ELM algorithm involves least squares which is extended to kernel learning framework [15]. Because of its excellent performance, ELM has been applied in various fields. In hyperspectral image processing field, Pal et al. [16] applied ELM based on kernel to classify remote sensing image, and it gives a better result than support vector machine (SVM) and some other neural network frameworks [17]. However, ELM execution speed is far less than SVM. Bazi et al. [18] selected different algorithm for the optimal classification parameters of ELM based on kernel function.

The main contributions of this paper are as follows:

- To solve the problem of hyperspectral remote sensing images data classification, this paper proposes a classification algorithm based on improved Rotation Forest, namely ROF-KELM.
- To get effective remote sensing data characteristics, the proposed algorithm uses NMF to do feature extraction due to the non-negative characteristics of remote sensing image data.
- To get high diversity among the base classifiers, the proposed algorithm uses Q-statistic to select base classifiers.
- This paper uses AVIRIS image data, ROSIS image data and UCI data sets to do experiment to evaluate the performance of ROF-KELM, and compares with some existing neural network ensemble algorithms. The proposed algorithm has higher classification accuracy and stronger generalization performance.

The aforementioned facts motivated us to develop a novel hyperspectral remote sensing images classification method. The rest of this paper is organized as follows. Section 2 briefly surveys related work. Section 3 presents a brief review of several related algorithms and gives the details of the proposed ROF-KELM algorithm. Section 4 illustrates two examples, including hyperspectral remote sensing image data and UCI data classification, to show the excellent performance of proposed ROF-KELM algorithm. Finally, discussions and conclusions are given in Section 5.

## 2. Related Work

The development of IoT plays a vital role in remote sensing image classification technology [19]. Several ensemble techniques for classification in the remote sensing imagery have been proposed till now. Borja et al. [20] proposed a new semisupervised segmentation algorithm for hyperspectral image segmentation. Du et al. [21] had applied firstly Rotation Forest to the classification of hyperspectral remote sensing image. At the same time, to overcome the shortage of ELM, Du et al. [22] proposed Bagging-based and AdaBoost-based ELMs. Bao et al. [23] proposed a new rotation forest algorithm based on weight for the classification of hyperspectral remote sensing image. Li et al. [24] gave a brief overview of typical deep learning models, and it shows a systematic review of pixel-wise and scene-wise remote sensing image classification approaches that are based on use of deep learning.

Zhou et al. [25] proposed a new NMF algorithm based on region structure to explore consistent data distribution in the same region while distinguishing different data structures across regions in the no-mixed data. Tsinos et al. [26] proposed a novel unmixing method that is based on a simultaneously spare and low-rank constrained NMF. To linear hyperspectral unmixing, Wang et al. [27] proposed a novel Group NMF method based on group low-rank constrain, combining the low rank prior of hyperspectral data with semantic information. Karoui et al. [28] proposed two new methods, related to linear spectral unmixing techniques, and based on NMF, optimizing a new joint NMF method. Zhang et al. [29] proposed a new algorithm about dimension reduction of hyperspectral data based on non-negative discriminative manifold learning, which yields a discriminative and low dimensional feature representation.

Mujica et al. [30] explored the use of principal component analysis and $T^{2}$ and Q-statistic measures to detect and distinguish damages in structures. Ansari et al. [31] reported about Q-statistic concept to improve the performance of generalized differences algorithm based on intensity histogram for imaging functional blood vessel structures in a rodent window chamber of a mice. Rabal et al. [32] introduced Q-statistic concepts to improve the performance of some methods based on the histogram to estimate dynamic speckle activity.

Wu et al. [33] proposed a novel multiple features fusion method for remote sensing image classification based on ELM. Weng et al. [34] proposed a classification method based on deep learning, which combines convolutional neural networks and ELM to improve classification performance. Han et al. [35] proposed a remote sensing image classification algorithm using stacked autoencoder and ensemble of ELM named SAE-ELM.

## 3. Proposed Learning Algorithm

In this section, we describe the basic algorithm, include Rotation Forest, NMF, ELM, KELM, Q-statistic and the proposed algorithm.

### 3.1. Rotation Forest

Let $X={\left[x_{1},\ldots ,x_{n}\right]}^{T}$ be a training sample characterized by *n* features and *X* as the training sample data of an N×n matrix. Let $Y={\left[y_{1},\ldots ,y_{N}\right]}^{T}$ be as the class labels. Denote by $\Gamma_{1},\ldots ,\Gamma_{L}$ the classifier in the ensemble, and *F* is the feature set. The steps for training classifier $\Gamma_{i},i=1,\ldots ,L$ are handled in the following.

*F* is split into *K* feature sets and each subset contains M=n/K number of features. Let $F_{i,j}$ be the *j*th,1,…,K subset of features for $\Gamma_{i}$, and $X_{i,j}$ be the features in $F_{i,j}$ frame. $X_{i,j}^{\prime }$ is denoted as a new training set which is selected from $X_{i,j}$ randomly using bootstrap algorithm. Then, we transform $X_{i,j}^{\prime }$ to get the coefficients $a_{i,j}^{\left(1\right)},\ldots ,a_{i,j}^{\left(M\right)}$, the size of $a_{i,j}^{\prime }$ is M×1. A spare rotation matrix $R_{i}$ is organized with the above coefficients

(1) $$R_{i}=\left[\begin{array}{cccc} a_{i,1}^{\left(1\right)},\ldots ,a_{i,1}^{\left(M_{1}\right)} & 0 & \ldots & 0\\ 0 & a_{i,2}^{\left(1\right)},\ldots ,a_{i,2}^{\left(M_{2}\right)} & \ldots & 0\\ \vdots & \vdots & \ddots & \vdots \\ 0 & 0 & \ldots & a_{i,k}^{\left(1\right)},\ldots ,a_{i,k}^{\left(M_{k}\right)} \end{array}\right]$$

where $R_{i}$ is rearranged to $R_{i}^{a}$ with respect to the original feature set. Then, the training set will become $XR_{i}^{a}$. In this case, all classifiers will be trained in parallel style. For a given test sample χ, the confidence is calculated for each class by the average combination method

(2) $$\mu_{k}\left(\chi \right)=\frac{1}{L}\sum_{i=1}^{L}\gamma_{i,k}\left(\chi R_{i}^{a}\right)k=1,\ldots ,c$$

where $\gamma_{i,k}\left(\chi R_{i}^{a}\right)$ is the probability generated by the classifier $\Gamma_{i}$, suppose that χ belongs to class *k*.

Finally, χ is the class with the largest confidence. It selects the sample size $X_{i,j}$ bigger than $X_{i,j}^{\prime }$, and aims at two aspects as follows:

- Avoid obtaining the same coefficients of the transformed components if the same features are chosen.
- Enhance the diversity among the generated ensemble base classifiers.

### 3.2. Non-Negative Matrix Factorization

Remote sensing image data has non-negative characteristics. When we deal with these data in a linear notation, the decomposition must be non-negative. In this case, if we adopt a method PCA in the Rotation Forest system, some physical properties are lost which leads to the result negative. So it is effective to avoid this problem by using non-negative matrix factorization.

As a matrix decomposition algorithm, NMF gives non-negative constraints to every value in the processing matrix. Set *Q* be a M×N matrix. Then decompose *Q* into *W* and *H*, *W* and *H* are non-negative:

(3) Q≈WH

where *W* is a M×T matrix as basic matrix and *H* is a T×N matrix as coefficient matrix. When *M* is bigger than *T*, to get the dimension reduction, the coefficient matrix can be selected instead of the original data matrix. At the same time, due to the non-negative constrains of every value during the decomposition, there are additive joints.After decomposition, *W* and *H* make the feature information of the original matrix *Q* well.

### 3.3. Extreme Learning Machine

**Figure 1:**
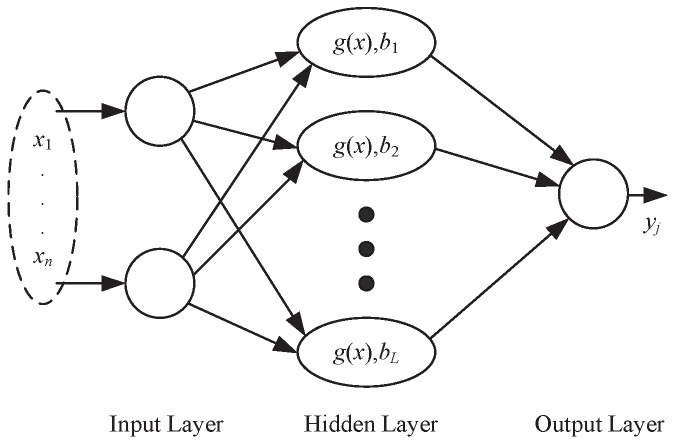
Classification results by different algorithms on AVIRIS image data.

Extreme learning machine (ELM) is a new feedforward neural network training paradigm, where a non-iteration learning method is performed. Commonly, ELM consists of input layer, hidden layer, and output layer. Figure 1 illustrated the single-hidden-layer structure of ELM. The hidden layer building process is the most different between ELM and traditional neural networks. There are usually much more nodes in ELM’s hidden layer than in traditional neural networks. Meanwhile, the beginning of training, the input weights and hidden layer biases of ELM are determined randomly and keep fixed during training process. The output weights of ELM are the only tunable weights and simple linear regression can get satisfying results. The mathematic equation of ELM is summarized as follows:

(4) $$\sum_{i=1}^{L}w_{i}g(W_{in(i)},b_{i},x_{j})=\sum_{i=1}^{L}w_{i}g\left(W_{in(i)}\cdot x_{j}+b_{i}\right)=y_{j},j=1,\ldots ,N$$

where *L* is the size of the hidden neurons, $x_{j}\in R^{n}$ denotes the input vector, $y_{j}\in R$ denotes the output (only scalar case is considered in this equation), $W_{in(i)}\in R^{n}$ is the input weights vector responding to the *i*th hidden node, $W_{in(i)}\cdot x_{j}$ denotes the inner product of $W_{in(i)}$ and $x_{j}$, $b_{i}\in R$ denotes the bias value of the *i*th hidden neuron, g(·) denotes the activation function (sigmoid function is usually used), $w_{i}\in R$ denotes the output weight value corresponding to the *i*th hidden node and *N* denotes the size of the training samples. At the very beginning, the input weights $W_{in}$ and the bias *b* are randomly valued and keep fixed in the learning procedure.

Equation (4) can be rewritten compactly as follows:

(5) Hw=y.

where

$$H={\left[\begin{array}{ccc} g(W_{in(1)},b_{1},x_{1}) & \ldots & g(W_{in(L)},b_{L},x_{1})\\ \vdots & \ddots & \vdots \\ g(W_{in(1)},b_{1},x_{N}) & \cdots & g(W_{in(L)},b_{L},x_{N}) \end{array}\right]}_{N\times L}$$

$w={\left[w_{1},w_{2},\cdots ,w_{L}\right]}^{T}$, $y={\left[y_{1},\ldots ,y_{N}\right]}^{T}$ denotes the output of ELM, g(·) denotes the sigmoid activation function, $b_{i}\in R$ denotes the bias value of the *i*th hidden neuron and *H* is named the hidden layer output matrix.

Suppose the ELM consisting of *L* nonlinear processing hidden nodes can learn the training dataset (the size is *N*) correctly, so that there are $w_{i},i=1,\ldots ,N$ to make the following Equation (6) hold.

(6) $$\sum_{i=1}^{L}w_{i}g\left(W_{in(i)}\cdot x_{j}+b_{i}\right)=t_{j},j=1,\ldots ,N.$$

where $t_{j}$ is the target value, and *N* is the number of training samples.

To simplify computation, Equation (6) can be written as follows:

(7) Hw=t

where $t={\left[t_{1},\ldots ,t_{N}\right]}^{T}$ is the target vector. Since the input weights $W_{in)}$ and the bias *b* have been randomly determined before the learning process, Equation (7) essentially can be seen as a linear regression problem, and the smallest norm least squares solution of Equation (7) is

(8) $$w=H^{†}t$$

where $H^{†}$ denotes the Moore-Penrose generalized inverse of the hidden layer output matrix *H*.

The hyperparameter of ELM that should be determined empirically is the number of the hidden nodes.

### 3.4. Kernel Extreme Learning Machine

When the feature mappings of ELM are unknown to users, that is a kernel trick is conducted, kernel extreme learning machine (KELM) is developed, and the simulation results indicate that KELM can achieve similar or better generalization performance with much faster learning speed than traditional SVR. Using Equation (8), the output weights of ELM can be calculated one shot, avoiding the iteration of gradient decent. Nevertheless, the structure of ELM, namely the size of hidden layer that is a hyper-parameter that has very important effect on the learning performance, is very hard to choose the optimal value in a specific learning environment. Furthermore, support vector regression (SVR), as the representative of kernel methods, where kernel tricks are applied to do the inner product, are widespread used in many date processing fields. Generally speaking, ELM and SVR both are variants of single-hidden-layer feedforward network. As a result, some researchers have been studying the relationships between ELM and SVR. Without structure determination, Kernel Extreme Learning Machine (KELM) is proposed. Hereafter, we employ the expression $\phi \left(x\right)$ in place of $h\left(x\right)$ to explicitly indicate that the hidden layer mapping can be unknown.Consequentially, the kernel matrix of ELM is written as:

(9) $$K_{ELM}\left(x,x^{\prime }\right)=\phi \left(x\right)\cdot \phi \left(x^{\prime }\right)$$

The output function $f\left(x\right)$ of KELM is formulated as:

$$\begin{array}{l} \;f\left(x\right)=\phi \left(x\right)H^{T}{\left(\frac{I}{C}+HH^{T}\right)}^{-1}t\\ ={\left[\begin{array}{c} K_{ELM}\left(x\cdot x_{1}\right)\\ \vdots \\ K_{ELM}\left(x\cdot x_{N}\right) \end{array}\right]}^{T}{\left(\frac{I}{C}+K_{ELM}\right)}^{-1}t \end{array}$$

where *C* is regularization coefficient.

The unknown hidden layer mapping ϕ(·) of KELM is very similar to that of SVR, and the same as SVR, the kernel $K_{ELM}\left(\cdot ,\cdot \right)$ should be declared. As a result, the structure of ELM is no longer need to determine. It is assumed to have the training set $T=\left(x_{i},t_{i}\right),i=1,\ldots ,N$, where $x_{i}\in R^{n}$, and $t_{i}\in R$.

The corresponding Lagrangian dual optimization problem of Equation (10) is:

(10) $$L_{D}=\frac{1}{2}{∥w∥}^{2}+\frac{C}{2}\sum_{i=1}^{N}\xi_{i}^{2}-\sum_{i=1}^{N}\alpha_{i}\left(\phi \left(x_{i}\right)w-t_{i}+\xi_{i}\right)$$

where $\alpha_{i}$ is the *i*th Lagrangian multiplier. The optimality conditions of Equation (10) can be formulated as:

(11) $$\frac{\partial L_{D}}{\partial w}=0\to w=\sum_{i=1}^{N}\alpha_{i}\phi \left(x_{i}\right)$$

(12) $$\frac{\partial L_{D}}{\partial \xi_{i}}=0\to \alpha_{i}=C\xi_{i},i=1,\ldots ,N$$

(13) $$\frac{\partial L_{D}}{\partial \alpha_{i}}=0\to w^{T}\phi \left(x_{i}\right)-t_{i}+\xi_{i}=0,i=1,\ldots ,N$$

By substituting Equations (11) and (12) into Equation (13), we can get

(14) $$\alpha ={\left(K_{ELM}+\frac{I}{C}\right)}^{-1}t$$

where $\alpha ={\left[\alpha_{1},\ldots ,\alpha_{N}\right]}^{T}$, $K_{ELM(i,j)}=\phi \left(x_{i}\right)\cdot \phi \left(x_{j}\right);\;i,j=1,\ldots ,N$. Hence, the corresponding output function is:

(15) $$f\left(x^{\prime }\right)=K_{ELM}\left(x^{\prime },x\right){\left(K_{ELM}+\frac{I}{C}\right)}^{-1}t$$

Equation (10) can be transformed to the following expression:

(16) $$L_{D}=\alpha^{T}t-\frac{1}{2}\alpha^{T}K_{ELM}\alpha -\frac{1}{2C}\alpha^{T}\alpha$$

The same as kernel method, the type of kernel function and the corresponding kernel parameters of KELM should be carefully determined and there are no theoretical guides. Simultaneously, the hyperspectral image has complex spatial and spectral information, the represented capacity of a single kernel may not enough.

### 3.5. Q-Statistic

Given *N* training samples, suppose there are two classifiers $C_{i},\;C_{j}$, $N^{11}$ and $N^{00}$ are the number of samples with correct classification and wrong classification of $C_{i}$ and $C_{j}$ respectively, $N^{10}$ is the number of samples with $C_{i}$ correct classification and $C_{j}$ wrong classification, $N^{01}$ is the number of samples with $C_{j}$ correct classification and $C_{i}$ wrong classification. The Q-statistic of $C_{i}$ and $C_{j}$ as $Q_{i,j}$ is defined as:

(17) $$Q_{i,j}=\frac{N^{11}N^{00}-N^{01}N^{10}}{N^{11}N^{00}+N^{01}N^{10}}$$

It can be seen from Equation (9) that the value of $Q_{i,j}$ is between −1 and 1. If the two classifiers are independent of each other, the value of $Q_{i,j}$ is 0. If the two classifiers tend to divide one target correctly into the same class, the value of $Q_{i,j}$ is positive. If two classifiers tend to divide a target into the same class, the value of $Q_{i,j}$ is negative. If there are *k* classifiers, the Q-statistic mean of the pair classifier as $Q_{av}$ is shown in Equation (10):

(18) $$Q_{av}=\frac{2}{k(k-1)}\sum_{i=1}^{k-1}\sum_{j=i+1}^{k}Q_{i,j}$$

Q-statistic can better measure the differences between the base classifiers in the integration algorithm, and the calculation is simple. Therefore, the proposed algorithm selects the Q-statistic as a measure index to obtain better classification results when selecting a large difference base classifier.

### 3.6. ROF-KELM Algorithm

**Figure 2:**
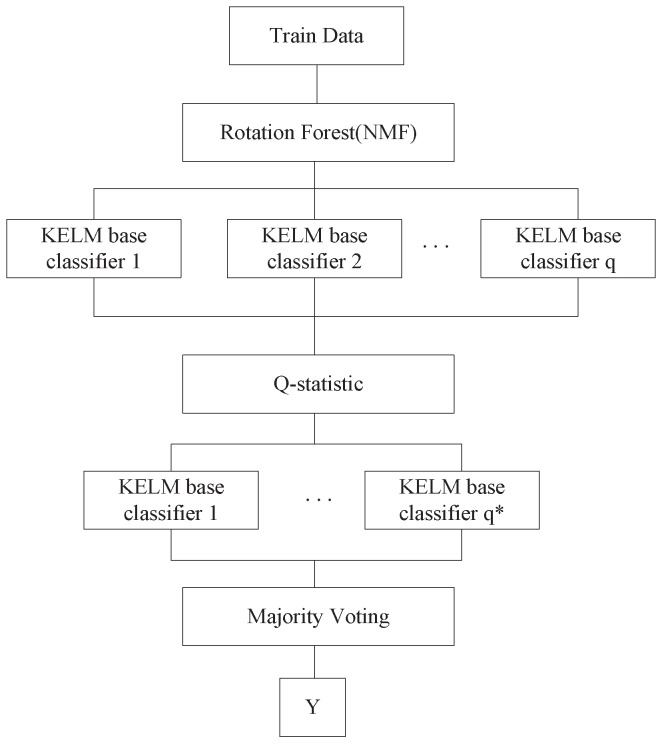
The structure of ROF-KELM.

KELM as base classifier is used in Rotation Forest algorithm and then using NMF to replace PCA for extraction and become a new ensemble algorithm, which is called ROF-KELM. It improves diversity to get better classification result. The structure of ROF-KELM is show in Figure 2.

Set $p={\left[p_{1},p_{2},\ldots ,p_{n}\right]}^{T}$ be sample points described by *n* features. Set *P* be sample points set containing the training data as a n×N matrix. Set $Y=[y_{1},y_{2},\ldots ,y_{N}]$ be a vector with class labels, where $y_{1}$ takes a value from the set of class labels $\left\{l_{1},l_{2},\ldots ,l_{c}\right\}$ and *c* is the number of labels. Denote by $B_{1},B_{2},\ldots ,B_{n}$ base classifier number, ROF-KELM is described as belows:

- Step (1) Divide the sample into two parts, 80% of the sample as training data *P*, 20% as test data.
- Step (2) Select a bootstrap sample from *P*.
- Step (3) Apply NMF on the training data in order to obtain the coefficient matrix.
- Step (4) Arrange and re-order the NMF resulted coefficient matrix to obtain the rotation matrix, according to the sequence of *Q*.
- Step (5) Calculate the hidden layer output matrix *H* using the initial kernels.
- Step (6) Calculate the output weight β, where $\beta =H^{†}T$.
- Step (7) Use the kernel function to train ELM.
- Step (8) Calculate Q-statistic through Equation (18), selected base classifiers are the final base classifier sets, which number is $q^{*}$.
- Step (9) Use majority voting method for final base classifiers to obtain the final classification results.

## 4. Simulation Results

In this section, we will give two examples to substantiate the proposed ROF-KELM for hyperspectral image classification. First, ROF-KELM is used to classify a hyperspectral image and the result is compared with some state-of-the-art classification methods. Then, ROF-KELM is tested on UCI data classification example to demonstrate the superior performance.

### 4.1. Simulation Results for AVIRIS Data Set

**Figure 3:**
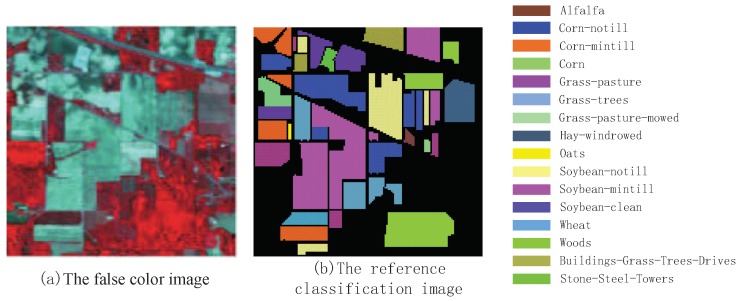
The schematic diagram of AVIRIS image.

To verify the performance of ROF-KELM algorithm, we did an experiment using hyperspectral remote sensing data called AVIRIS obtained from the airborne visible infrared imaging spectroradiometer of NASA. It is from an Indian Pines forest test site in northwestern Indiana, USA. The image contains 145×145 pixels, with 200 spectral bands (104–108, 150–163, and 220). The spatial resolution is 20 m/pixel. The classification data of AVIRIS is shown in Table 1. The schematic diagram of AVIRIS image is shown in Figure 3.

The kernel function of KELM uses Gauss kernel $K\left(u,v\right)=exp\left(-\gamma {∥u-v∥}^{2}\right)$. The kernel width is set to 10. The regularization parameter is also set to 10. 80% of training samples are used for training models, and the remaining 20% are used as test samples. They are used to determine the number of ensemble KELM, it generates 20 base classifiers each time for selective ensemble. Based on Q-statistic, it is determined that the number of base classifiers is 8, and gets maximum diversity. The simulation of the all algorithms on the dataset is carried out using MATLAB 2016a on a machine with an Intel Core i7, 2.26 GHz, 4 Cores CPU and 4 GB RAM.

In these experiments, the classification accuracies for the proposed algorithm are obtained and evaluated as shown in Table 2. The overall classification accuracy(OA) is the ratio of the number of pixel categories to the total number of categories. The Kappa coefficient (Kappa) is the ratio of error reduction produced by classification and completely random classification. The value of OA and Kappa from the proposed ROF-KELM algorithm have reached 0.9457 and 0.9322, better than the comparing algorithms of Bagging [36], Random Forest [37], Rotation Forest [13], SVM [22] and KELM [16]. Similarly, the high overall classification accuracy indicates that the algorithm has good effects in classifying AVIRIS images, and the high Kappa coefficient indicates good stability of the algorithm. Therefore, the proposed ROF-KELM algorithm performs well in the classification processing of AVIRIS images.

**Figure 4:**
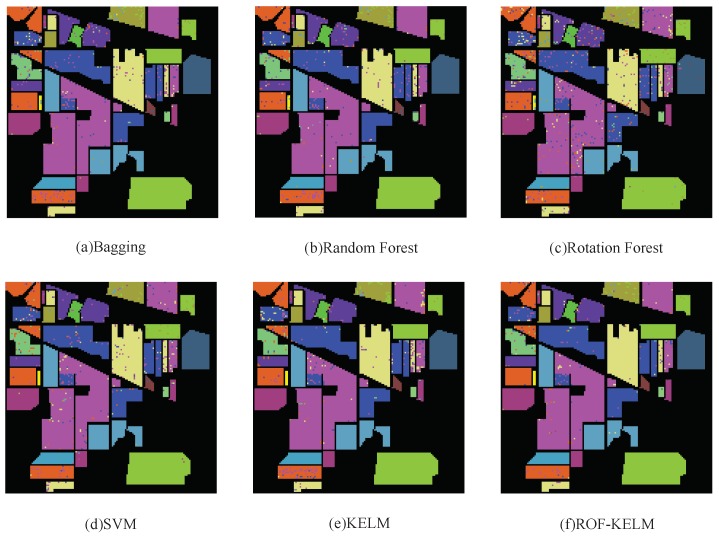
Classification results by different algorithms on AVIRIS image data.

Eighty percent of all sample data was used as training data to classify the whole image. The classification figure is shown in Figure 4. From the classification results of 6 algorithms, it can be seen that ROF-KELM algorithm has obvious advantages over the other 5 algorithms in classifying class 10 and class 11. In contrast to class 10, it can be seen that ROF-KELM classification result has the least wrong sample points. Rotation Forest classification result has the most wrong sample points. In contrast to class 11, it can be seen that the classification result of Bagging, Random Forest and ROF-KELM are relatively sparse, and the error sample points of the classification result of KELM are denser in the small area. From the number of error sample point, the error rate of ROF-KELM algorithm is the lowest. The advantages of the other categories are not particularly obvious. From the result analysis, the spatial information of class 10 and class 11 is more suitable for ROF-KELM algorithm.

### 4.2. Simulation Results for ROSIS Data Sets

**Figure 5:**
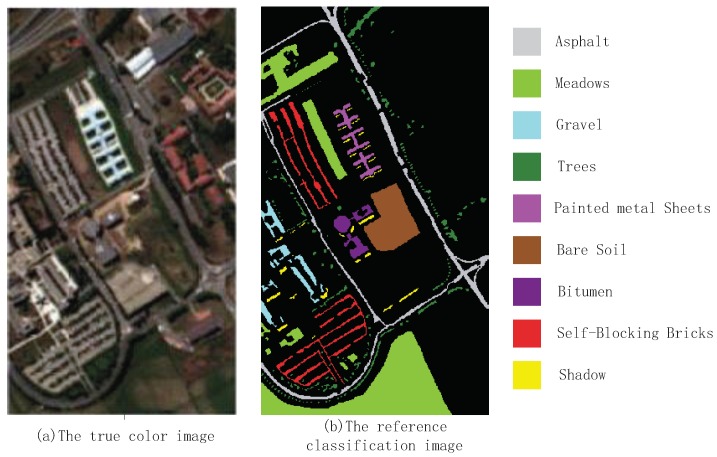
The schematic diagram of ROSIS image.

The second hyperspectral remote sensing image is about the University of Pavia remote sensed by Reflective Optices System Imaging Spectrometer(ROSIS). It exists 115 spectral bands across 0.43 to 0.86 μm spectral range in the original hyperspectral remote sensing image. After preprocessing, 12 bad bands are removed and 103 bands are remained in this simulation. The University of Pavia image consists of pixels and the spatial resolution of is 1.3 m per pixel. There are 9 ground-truth classes in the University of Pavia image, and 42776 samples are labeled. The details of the University of Pavia image labeled samples are shown in Table 3. From Table 3, There are about more than 1000 samples for every class of the University of Pavia image. The schematic diagram of ROSIS image is shown in Figure 5.

In these experiments, the classification accuracies for the proposed algorithm are obtained and evaluated as shown in Table 4. The classification overall accuracy and Kappa coefficient from the proposed ROF-KELM algorithm have reached 0.9524 and 0.9351, better than the comparing algorithms of Bagging, Random Forest, Rotation Forest, SVM, and KELM. The high overall classification accuracy indicates that the algorithm has good effects in classifying ROSIS images, and the high Kappa coefficient indicates good stability of the algorithm. Therefore, the proposed ROF-KELM algorithm performs well in the classification processing of ROSIS images.

**Figure 6:**
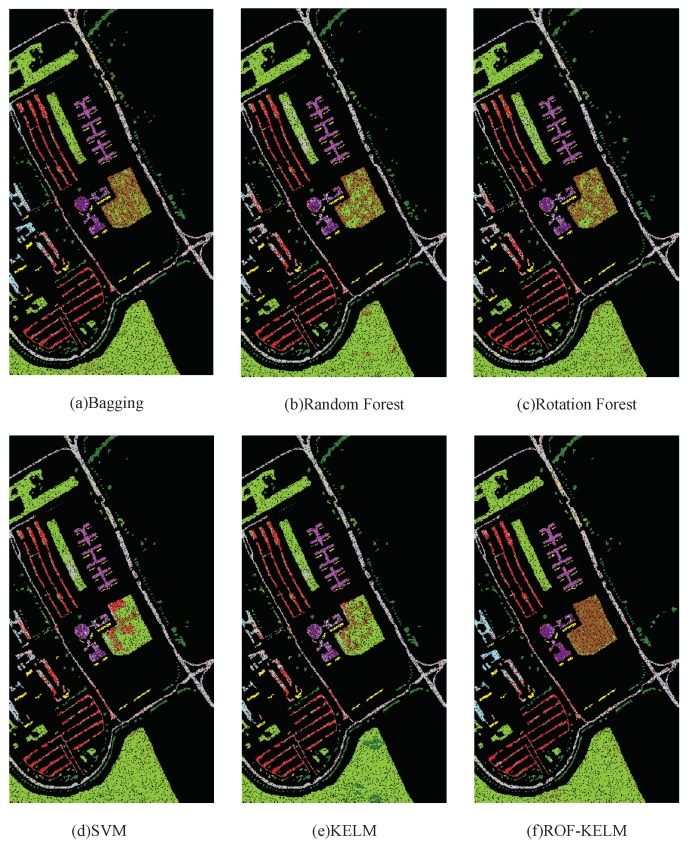
Classification results by different algorithms on ROSIS image data.

As it can be seen from Figure 6, compared to class 2, the KELM algorithm has a large density of local error data points. It can be seen that the classification effect of KELM algorithm to class 2 data is not as good as the other 5 algorithms. Compared to class 6, it can be seen that KELM and SVM algorithms have a large range of data points wrong into class 2 data, and the over fitting is serious and does not get good expected results, while KELM and ROF-KELM algorithm also have local error, and the error rate is low, and the comparison can be seen that the classificaiton efficiency of ROF-KELM to class 6 is better than the other algorithms. From the whole sample classification, compared with the other 5 algorithms, ROF-KELM algorithm is more robust in the data processing in each class, and is more adaptable to different types of data, and the result of higher precision are obtained.

### 4.3. Simulation Results for UCI Data Sets

To further verify the effectiveness of the proposed algorithm ROF-KELM, which is compared to the UCI databases [38], and 4 sets of UCI data are selected. The properties of each group are shown in Table 5. In the experiment, ROF-KELM is compared with the classical algorithm Bagging [36], Adaboost [12] and Rotation Forest [13] respectively, the parameter selection is the same as the experiment A, the results are shown as Table 6.

As can be seen from Table 6, the overall accuracy of the proposed algorithm reaches respectively 0.9239, 0.8952, 0.8325, 0.7891. It has the highest accuracy in the 4 sets of UCI data. It shows that the generalization performance of the proposed algorithm is stronger. It not only has good classification results for hyperspectral remote sensing data, but also has good classification results for many data with many dimensions and classes.

## 5. Conclusions

An improved Rotation Forest and KELM named ROF-KLEM is proposed in this paper. This algorithm uses Rotation Forest to segment the original training set, and then the sub feature is transformed by NMF to improve the difference between the training data. KELM as base classifier is used to train the model. And then, Q-statistic is used to measure the classifiers between the various base classifiers. The difference between the training data and between base classifiers are chosen to integrate the results, and finally, the voting results are used to get the final classification results. The performance of the proposed method has been tested by three examples, which are AVIRIS image, ROSIS image and the UCI data sets. The simulation results indicate the effectiveness of the proposed improve Rotation Forest algorithm.

## Figures and Tables

**Table 1:** The ground category and sample number of AVIRIS image data.

	Class	Samples
1	Alfalfa	46
2	Corn-no till	1428
3	Corn-mintill	830
4	Corn	237
5	Grass-pasture	483
6	Grass-trees	730
7	Gras-pasture-mowed	28
8	Hay-windrowed	478
9	Oats	20
10	Soybean-no till	972
11	Soybean-min till	2455
12	Soybean-clean	593
13	Wheat	205
14	Woods	1265
15	B-G-Trees-Drives	386
16	S-Steel-Towers	93

**Table 2:** The OA and Kappa comparison of AVIRIS image data classification.

	OA	Kappa
Bagging [36]	0.8787	0.8420
Random Forest [37]	0.8576	0.8366
Rotation Forest [13]	0.7569	0.7239
SVM [22]	0.8794	0.8628
KELM [16]	0.9136	0.9013
ROF-KELM	0.9457	0.9322

**Table 3:** The ground category and sample number of ROSIS.

	Class	Simples
1	Asphalt	6631
2	Meadows	18,649
3	Gravel	2099
4	Trees	3064
5	Painted metal Sheets	1435
6	Bare Soil	5029
7	Bitumen	1330
8	Self-Blocking Bricks	3682
9	Shadows	947

**Table 4:** The OA and Kappa comparison of ROSIS image data classification.

	OA	Kappa
Bagging [36]	0.9033	0.8872
Random Forest [37]	0.8802	0.8624
Rotation Forest [13]	0.8914	0.8728
SVM [22]	0.8542	0.8534
KELM [16]	0.8671	0.8492
ROF-KELM	0.9524	0.9351

**Table 5:** The feature of 4 UCI data.

	Instances	Attributes	Labels
Balance scale	625	4	3
Zoo	101	17	7
Flag	194	29	6
Pima Indians Diabetes	768	8	2

**Table 6:** The overall accuracy of UCI data sets.

	Bagging [36]	Adaboost [12]	Rotation Forest [13]	ROF-KELM
Balance scale	0.7832	0.7442	0.8200	0.9239
Zoo	0.8118	0.7162	0.7623	0.8952
Flag	0.6900	0.6152	0.4627	0.8325
Pima Indians Diabetes	0.7566	0.7344	0.6720	0.7891
